# Photonic matrix multiplication lights up photonic accelerator and beyond

**DOI:** 10.1038/s41377-022-00717-8

**Published:** 2022-02-03

**Authors:** Hailong Zhou, Jianji Dong, Junwei Cheng, Wenchan Dong, Chaoran Huang, Yichen Shen, Qiming Zhang, Min Gu, Chao Qian, Hongsheng Chen, Zhichao Ruan, Xinliang Zhang

**Affiliations:** 1 Wuhan National Laboratory for Optoelectronics, Huazhong University of Science and Technology, Wuhan, 430074 China; 2 Department of Electronic Engineering, The Chinese University of Hong Kong, Shatin, Hong Kong, China; 3 Lightelligence, Hangzhou, 311121 China; 4 Institute of Photonic Chips, University of Shanghai for Science and Technology, Shanghai, 200093 China; 5 Centre for Artificial-Intelligence Nanophotonics, School of Optical-Electrical and Computer Engineering, University of Shanghai for Science and Technology, Shanghai, 200093 China; 6 Interdisciplinary Center for Quantum Information, State Key Laboratory of Modern Optical Instrumentation, ZJU-Hangzhou Global Scientific and Technological Innovation Center, ZJU-UIUC Institute, Zhejiang University, Hangzhou, 310027 China; 7 Interdisciplinary Center of Quantum Information, State Key Laboratory of Modern Optical Instrumentation, and Zhejiang Province Key Laboratory of Quantum Technology and Device, Department of Physics, Zhejiang University, Hangzhou, 310027 China

**Keywords:** Optoelectronic devices and components, Photonic devices, Integrated optics

## Abstract

Matrix computation, as a fundamental building block of information processing in science and technology, contributes most of the computational overheads in modern signal processing and artificial intelligence algorithms. Photonic accelerators are designed to accelerate specific categories of computing in the optical domain, especially matrix multiplication, to address the growing demand for computing resources and capacity. Photonic matrix multiplication has much potential to expand the domain of telecommunication, and artificial intelligence benefiting from its superior performance. Recent research in photonic matrix multiplication has flourished and may provide opportunities to develop applications that are unachievable at present by conventional electronic processors. In this review, we first introduce the methods of photonic matrix multiplication, mainly including the plane light conversion method, Mach–Zehnder interferometer method and wavelength division multiplexing method. We also summarize the developmental milestones of photonic matrix multiplication and the related applications. Then, we review their detailed advances in applications to optical signal processing and artificial neural networks in recent years. Finally, we comment on the challenges and perspectives of photonic matrix multiplication and photonic acceleration.

## Abstract (web summary)

This review summarizes the advances of photonic accelerators from the viewpoint of photonic matrix multiplication, providing a guidance for all-optical or optoelectronic-hybrid AI hardware chip system.

## Graphical abstract

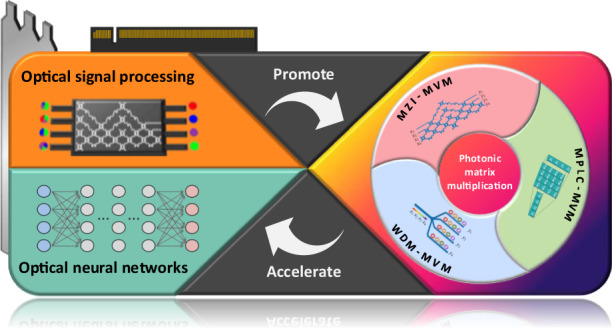

## Introduction

Over the past few years, there has been an ever-growing demand for artificial intelligence and fifth-generation communications globally, resulting in very large computing power and memory requirements. The slowing down or even failure of Moore’s law makes it increasingly difficult to improve their performance and energy efficiency by relying on advanced semiconductor technology^1,2^. Moreover, the clock frequency of traditional electrical processing methods is generally limited to several GHz^3^, which can no longer meet the demands of super-high-speed and low-latency mass data processing. Matrix computation is one of the most widely used and indispensable tools of information processing in science and engineering^4,5^. Most signal processing, such as the discrete Fourier transform and convolution operation, can be attributed to matrix computations. On the other hand, since the concept of artificial intelligence (AI) was put forward in 1956 for the first time^6^, artificial neural networks (ANNs) have been rapidly developed and widely used in various fields^7^. Due to the continuous substantial increase in information capacity, general electronic processors seem to be incapable of executing high-complexity AI tasks in the foreseeable future^1^. To solve this challenge, chips oriented to AI applications have emerged, such as neural network processing units (NPUs)^8^. At present, AI chips have been widely used in almost every type of big data processing in areas such as search, news, e-commerce, cloud computing, and inverse design of functional devices^9–13^. Typically, neural network algorithms represented by deep learning, such as forward neural networks (FNNs), convolutional neural networks (CNNs) and spiking neural networks (SNNs), are characterized by many training parameters, especially in heavy matrix computations^14^.

Traditionally, matrix computation is completed by an electrical digital signal processor, and its speed and power consumption are greatly limited by the nature of the electronic devices themselves. Therefore, traditional electrical methods are hard to simultaneously achieve high-capacity and low-latency matrix information processing limited by the Moore’s law^1,2^. However, for some applications, such as ultrafast neural networks^15^, large bandwidth and low latency are simultaneously required; thus, a new medium for matrix computations and interconnects is urgently needed for the implementation of high-performance and energy-efficient matrix computations. Optical devices can have a superlarge bandwidth and low power consumption^16^. And light has an ultrahigh frequency up to 100 THz and multiple degrees of freedom in their quantum state^17,18^, making optical computing one of the most competitive candidates for high-capacity and low-latency matrix information processing in the “More than Moore” era^1^. For example, a Fourier transform was performed at the speed of light with a lens^19^. Motivated by its very high prospect, photonic matrix multiplication has been developed rapidly in recent years and has been widely applied in photonic acceleration for optical signal processing^20–22^, AI and optical neural networks (ONNs)^15,23,24^. A lot of review works on photonic acceleration have been made, these works mainly focused on integrated photonic neuromorphic systems^1,15,23–28^, nanophotonics and machine learning blend^29,30^, reservoir computing^31^, programmable nanophotonics^21,22,32^. As a fundamental and important part of photonic acceleration, photonic matrix multiplication computation for photonic acceleration has not been systematically reviewed. Here, we review the advances of photonic acceleration from the perspective of photonic matrix multiplication. We first discuss the methods and developmental milestones of photonic matrix multiplications and then review the progress in cutting-edge fields of optical signal processing and optical neural networks. Finally, a perspective for photonic matrix multiplications is discussed.

## Matrix-vector multiplication

**Fig. 1:**
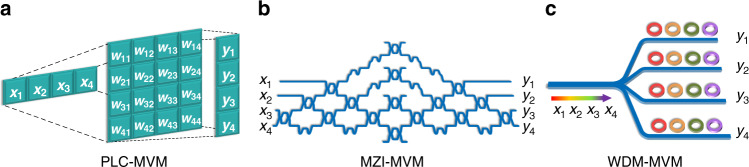
Methods for matrix multiplication computation. **a** PLC-MVM. **b** MZI-MVM. **c** WDM-MVM

The methods for photonic matrix-vector multiplications (MVMs) mainly fall into three categories: the plane light conversion (PLC) method, Mach–Zehnder interferometer (MZI) method and wavelength division multiplexing (WDM) method. The detailed mechanism of these MVMs can be found in ref. ^33^, which offers an easy-to-read overview of principle and development of photonic matrix computation. The first kind of optical MVM (PLC-MVM) is implemented by the diffraction of light in free space. Figure 1a shows a typical MVM configuration^34,35^. First, the incident vector of *X* distributed along the *x* direction can be expanded and replicated along the *y* direction through a cylindrical lens or other optical elements. Then, the spatial diffraction plane is used to adjust each element independently, and its transmission matrix is *W*. Finally, the *x*-direction beams are combined and summed in a similar way, and the final output vector of *Y* along the *y* direction is the product of the matrix of *W* and the vector *X*, that is, *Y =WX*. The second MVM mainly consists of an MZI network (i.e., MZI-MVM). Figure 1b shows the configuration diagram, which is based mainly on rotation submatrix decomposition and singular value decomposition^36^. The calibration of the transmission matrix is more difficult since every matrix element is affected by multiple dependent parameters. The third MVM (i.e., WDM-MVM) is an incoherent matrix computation method based on the WDM technology. Figure 1c shows a typical diagram based on microring resonators (MRRs). The input vector of *X* is loaded on beams with different wavelengths, which pass through the microrings with one-one adjustment of the transmission coefficients of *W*. Then, the total output power vector is given by *Y=WX*.

**Fig. 2:**
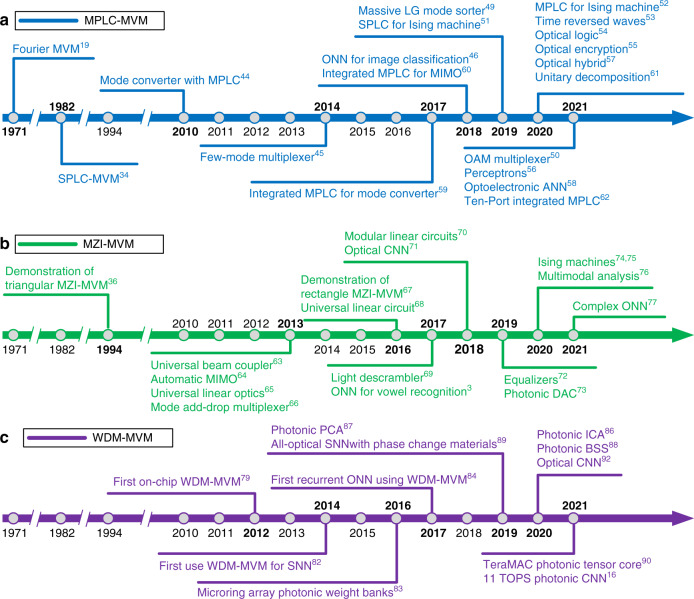
Timeline of advances in photonic matrix computations and neuromorphic photonics. **a** MPLC-MVM. **b** MZI-MVM. **c** WDM-MVM

Photonic matrix multiplication has come a long way and developed rapidly in recent years. Figure 2 summarizes the development history and milestones of photonic matrix computation. In the preliminary stage, only some fixed matrix computations were implemented using optical methods such as the Fourier transform^19^. Thereafter, the initially programmable MVM was demonstrated with spatial optical elements based on single PLC (SPLC)^34^. For example, a fully parallel, high-speed incoherent optical method was employed to utilize the discrete vector multiplier at a high speed^37^, while the update of the matrix at high frame rates was restrained with current spatial light modulators (SLMs). Matrix multiplications involving optical array modulators, such as electrooptic modulations, direct driven LED arrays, and acousto-optic Bragg cells, were accomplished with faster frame rates^34,38,39^. A photorefractive crystal^40–42^ and nonlinear material^43^ could be optionally applied to implement MVMs. In the SPLC-MVM method, only one dimension is used for the input/output vectors, and the scale ($\propto N$) of vectors is still limited. A more powerful PLC-MVM for unitary spatial mode manipulation was proved with multiplane light conversion (MPLC)^44,45^, in which the input/output vectors are distributed in the whole two-dimensional plane, and the scale is proportional to${N}^{2}$. Afterwards, the MPLC technique was widely used in various fields, such as for all-optical machine learning^46–48^, the Laguerre-Gaussian or orbital angular momentum (OAM) mode sorter^49,50^, the photonic Ising machine^51,52^, time-reversed optical waves^53^, optical logic operations^54^, optical encryption and perceptrons^55,56^, optical hybrid^57^ and neuromorphic optoelectronic computing^58^. Although MPLC can achieve ultralarge-scale MVMs, the devices are bulky, and the reprogramming speed for weight encoding is still limited. A mini-sized and universal MVM is more practical, especially in integrated photonic applications. In 2017, Tang et al. first proposed a novel integrated reconfigurable unitary optical mode converter using multimode interference couplers, which shared a similar principle with MPLC^59^. Then, it was used for all-optical on-chip multi-input-multi-output (MIMO) mode demultiplexing^60^. In 2020, the integrated MPLC technique was further analyzed by Saygin et al. as a novel matrix decomposition method based on multichannel blocks^61^ and then was experimentally proven on a silicon photonic chip^62^.

In 1994, Reck et al. proposed a recursive algorithm that could factorize any $N\times N$ unitary matrix into a sequence of two-dimensional matrix transformations, which paved the way for future photonic integrated computation based MZIs^36^. Then, Miller et al. suggested that the MZI network could be self-configured to define functions assisted by transparent detectors^63–66^. The MZI mesh was then applied in an add-drop multiplexer for spatial modes^66^, universal linear optical components^65^, automatic MIMO^64^ and universal beam couplers^63^. In 2016, Clements et al. proposed a brand-new universal matrix framework based on an alternative assemblage of MZIs and phase shifters, which is superior to that proposed by Reck et al. Only half the optical depth of the Reck design is required, and the optical loss is significantly reduced^67^. Ribeiro et al. experimentally demonstrated a 4 × 4-port universal optical linear circuit chip with the MZI mesh on integration platforms^68^. Thereafter, the applications of MZI-MVMs were further extended to ONNs^3^, light descramblers^69^, modular linear optical circuits^70^, optical CNNs^71^, equalizers^72^, digital-to-analog conversion (DAC)^73^, Ising machines^74,75^, mode analysis^76^ and complex ONNs^77^.

Generally, the footprint of the MZI reaches over 10,000 μm^2^ per interferometer unit, which remains a bottleneck to further improve the computing density of the MZI mesh. The WDM-MVM based on microring arrays was proposed by Xu et al., who used compact microrings with a diameter of only a few microns^78,79^. This approach encodes information on different optical wavelengths rather than spatial modes. Compared to other physical dimensions, the wavelength dimension has the most abundant orthogonal channels in optics, up to hundreds of channels^80,81^. Silicon MRR arrays for matrix operations were first conceptualized by Xu and Soref in 2011^78^. They were then demonstrated by Yang et al. using a 4 × 4 silicon microring modulator array but with binary values of 0 and 1 only^79^. In 2014, Tait and his colleagues proposed using MRR arrays as a matrix computation method primitive for photonic neural networks^82^ and achieved continuous matrix values from -1 to 1 by continuously tuning the MRRs. The WDM-MVM was further used for photonic weight banks^83–86^, principal component analysis (PCA)^87^, independent component analysis (ICA)^86^, blind source separation (BSS)^88^, TeraMAC neuromorphic photonic processor^18^, the optical SNN^89^, TeraMAC photonic tensor core^90^, optical CNN^91–93^, and photonic convolutional accelerator for the ONN^16,94^.

Table 1 summarizes the performance comparison of different photonic matrix multiplication methods. In general, the PLC-MVM method is coherent and can operate in the whole complex field. Its scale is very large, input vector sizes of 357 for SPLC-MVM^48^ and 490000 (*N* = 700) for MPLC-MVM^58^ were reported, easily up to 10^3^ for SPLC-MVM and 10^6^ for MPLC-MVM with SLMs^58^. However, the device size is quite large, and hence, the integrated counterpart was pursued^59–61^. The MZI-MVM method is also coherent, but its scale is far smaller than that of the PLC-MVM method (*N* = 64 was reported by Lightmatter^95^). The main advantage is that it can be integrated into a chip. The WDM-MVM method is more compact. The scale is restricted by the number of wavelengths and can be ~10^2^ with soliton crystal microcombs^16^, provided all the wavelengths are used for a single MVM. A balanced photodetector summing weighted signals allows for positive and negative weights^82^. WDM-MVM is incoherent and can be used for real-valued matrices. For these methods, the assigned transmission matrices for SPLC-MVM and WDM-MVM can be directly written in, while some algorithms are needed to load the transmission matrices for the MPLC-MVM and MZI-MVM methods. All these MVM methods have been widely applied in various fields. In the following, we review the detailed applications of MVMs in optical signal processing and photonic AI.

**Table 1:** Comparison of different photonic matrix multiplication methods

Method	Coherent computing	Integration	Input vector size	Matrix loading
SPLC-MVM	Yes	No	357 (ref. ^48^)	One-one
MPLC-MVM	Yes	Yes	40,000 (ref. 46) 490,000 (ref. ^58^)	Algorithm-aided
MZI-MVM	Yes	Yes	64 (ref. ^95^)	Algorithm-aided
WDM-MVM	No	Yes	~100 (ref. ^48^)	One-one

## MVMs for optical signal processing

The photonic matrix multiplication network itself can be used as a general linear photonic loop for photonic signal processing^32^. In recent years, MVM has been developed as a powerful tool for a variety of photonic signal processing methods.

### MPLC-MVMs

**Fig. 3:**
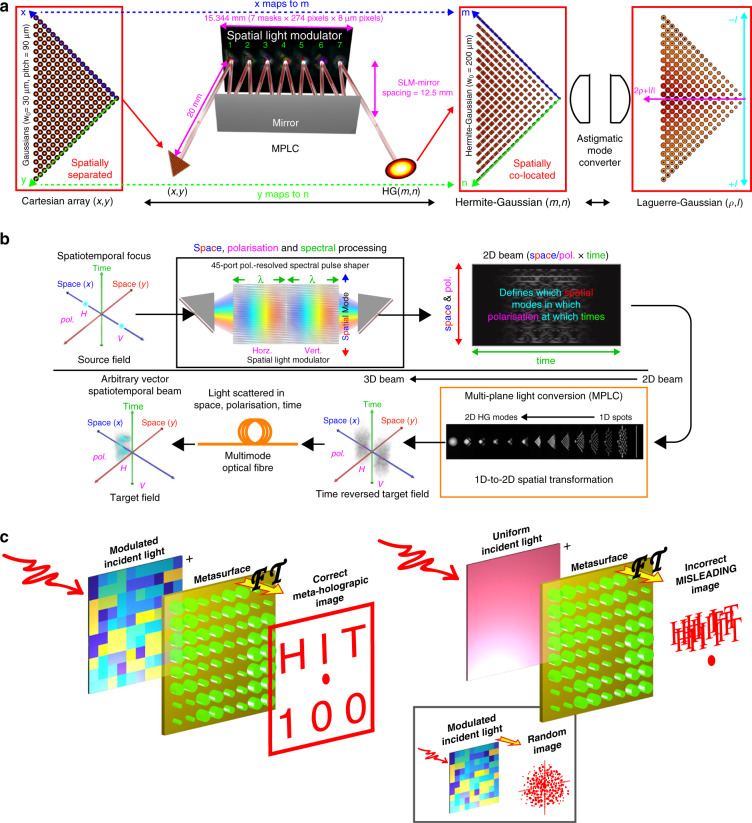
MPLC-MVMs for optical signal processing. **a** Laguerre-Gaussian mode sorter^49^. **b** Arbitrary vector spatiotemporal field generation^53^. **c** Optical encryption^55^. **a** Reprinted from ref. ^49^ with permission from Springer Nature: Nature Communications. **b** Reprinted from ref. ^53^ with permission from Springer Nature: Nature Communications. **c** Reprinted from ref. ^55^ with permission from Springer Nature: Nature Communications

Benefiting from the large-scale computing capability of spatial planes, MPLC can achieve very powerful matrix functions^44^. For example, Joel Carpenter et al. realized the classification of 210 Hermite–Gaussian modes or Laguerre-Gaussian modes using only 7 phase planes with a pixel size of $274\times 274$^49^. A schematic diagram of the Laguerre-Gaussian mode sorter is shown in Fig. 3a. First, Gaussian beams from different positions were injected into the device and converted to different orthogonal Hermite–Gaussian modes by MPLC based on the wavefront matching method^96^. Then, a cylindrical lens pair was used to convert the Hermite–Gaussian mode into the Laguerre-Gaussian mode. The realized super-multimode multiplexer and demultiplexer are of great significance in multimode optical communications. As shown in Fig. 3b, this powerful mode sorter was further used to create time-reversed waves, where all classical linear physical dimensions of light were simultaneously controlled independently^53^. This device can independently address the amplitude, phase, spatial mode, polarization and spectral/temporal degrees of freedom simultaneously through the programming of the SLM. Ninety spatial/polarization modes controlled over 4.4 THz at a resolution of ~15 GHz were demonstrated, covering a total of ~26,000 spatiospectral modes. A reprogrammable metahologram was further designed for optical encryption, as shown in Fig. 3c^55^. The encrypted information was divided into two matrices using two phase planes, and the enciphered message emerged only when the two planes matched.

**Fig. 4:**
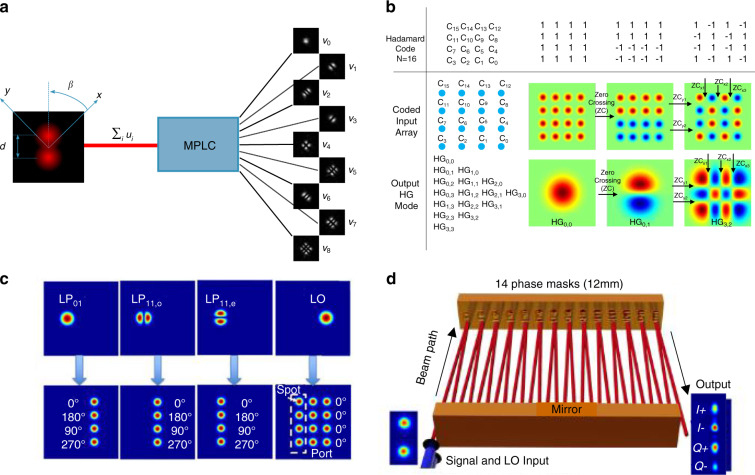
MPLC-MVMs for optical signal processing. **a** Transverse distance estimation^97^. **b** Nonmode selective Hermite–Gaussian mode multiplexer^98^. **c** Mode demultiplexing hybrid^100^. **d** Ultrabroadband polarization-insensitive optical hybrid^57^. **a** Reprinted with permission from ref. ^97^ © The Optical Society. **b** Reproduced from ref. ^98^ with the permission of Chinese Laser Press. **c** Reproduced from ref. ^100^ with the permission of Chinese Laser Press. **d** Reprinted from ref. ^57^ with the permission of IEEE Publishing

Some other applications have also been demonstrated. The MPLC technique was a helpful tool for optimal transverse distance estimation, as shown in Fig. 4a^97^. The measurements were performed in two dimensions far beyond the Rayleigh limit over a large dynamic range. Some theoretical studies were performed. For example, a scalable nonmode selective Hermite–Gaussian mode multiplexer was proposed, as shown in Fig. 4b, where 256 Hermite–Gaussian modes were designed using only seven phase masks^98^. In Fig. 4c, Li et al. implemented the linear polarization mode and Hermite–Gaussian mode demultiplexing hybrids with similar methods^99,100^. Each input mode was converted to four fundamental modes with a 90-degree phase difference located at nonoverlapping positions. Local light was uniformly mapped to the fundamental modes with the same phase, which exactly overlapped with output spots from the input modes. The complex amplitudes of the input modes could be retrieved from the interference light intensities. Furthermore, an ultrabroadband polarization-insensitive optical hybrid using MPLC was experimentally verified^57^. As shown in Fig. 4d, 14 phase masks and a gold mirror were employed to carry out the optical hybrid, and a measurement bandwidth of 390 nm was obtained.

**Fig. 5:**
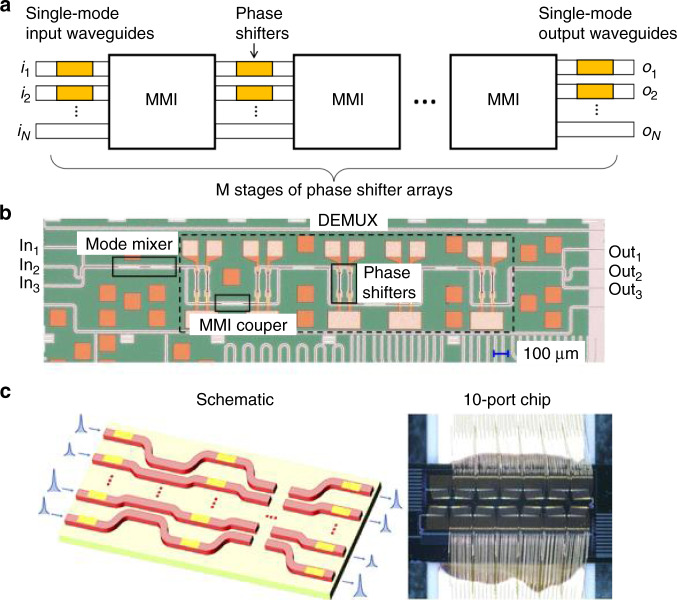
Integrated MPLC-MVMs. **a** Schematic diagram^59^. **b** Three-channel MIMO demultiplexing chip^60^. **c** Ten-port unitary optical processor^62^. **a** Reprinted from ref. ^59^ with the permission of IEEE Publishing. **b** Reprinted with permission from ref. ^60^ © The Optical Society. **c** Reproduced from ref. ^62^ with permission of ACS publications

Integrated MPLC-MVM was also successfully verified. In 2017, Tang et al. first theoretically proved a novel integrated reconfigurable unitary MPLC-MVM using multimode interference couplers^59^. The schematic diagram is presented in Fig. 5a. The transmission matrix was decomposed into a series of programmable unitary diagonal matrices and fixed unitary diffractive matrices. In theory, an arbitrary unitary transmission matrix can be configured by tuning the unitary diagonal matrices, provided that enough phase planes are assigned. In 2018, the integrated MPLC-MVM was experimentally verified for reconfigurable all-optical on-chip MIMO three-mode demultiplexing^60^. Figure 5b shows the details of the three-channel MIMO demultiplexing chip. Furthermore, Saygin et al. built a more universal architecture for integrated MPLC-MVM in 2020^61^. In addition, a ten-port unitary optical processor has been experimentally demonstrated^62^. Figure 5c presents the device operating principle, where the fixed unitary diffractive matrices are implemented using multiport directional couplers. This processer offers a new flexible and robust architecture for large-scale MVMs.

### MZI-MVMs

**Fig. 6:**
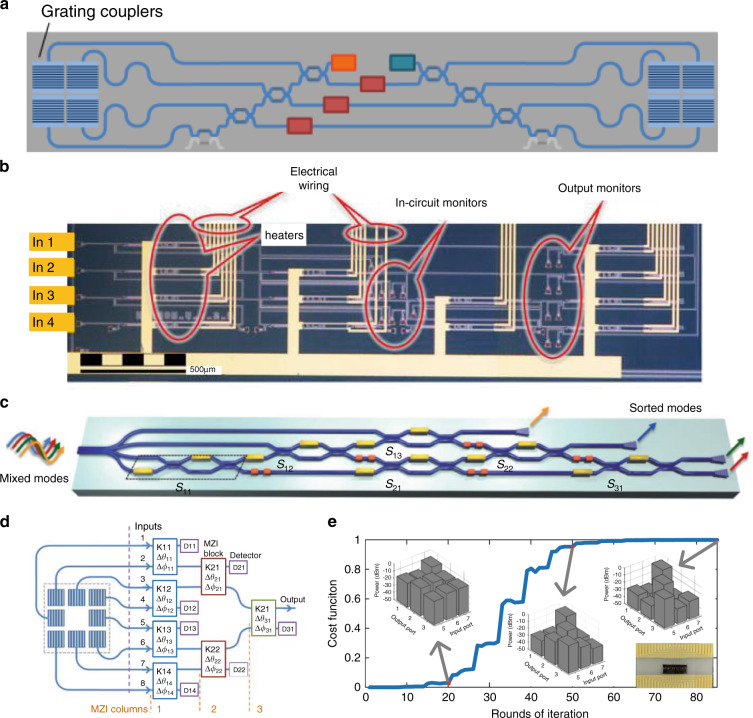
MZI-MVMs for optical signal processing. **a** Add-drop mode multiplexer^66^. **b** 4 × 4-port universal linear circuit^68^. **c** Optical mode descrambler^69^. **d** Analysis and generation of multimode optical fields^76^. **e** Self-configured example^103^. **a** Reprinted with permission from ref. ^66^ © The Optical Society. **b** Reprinted with permission from ref. ^68^ © The Optical Society. **c** Reproduced from ref. ^69^ with permission of Springer Nature: Light: Science & Applications. (d) Reprinted with permission from ref. ^76^ © The Optical Society. **e** Reprinted from ref. ^103^ with the permission of ACS Publishing

The MZI-MVM, as an integrated photonic matrix computation method, is quite suitable in on-chip optical signal processing^32,70^. Based on the orthogonal matrix transformation, it is competent to manipulate the spatial orthogonal modes. Figure 6a shows a reconfigurable add-drop multiplexer for spatial modes sampled by the grating array^66^. It could extract a specified spatial mode from a light beam, leaving the other modes undisturbed. It also allows a new signal to be reloaded on that mode. Similarly, as Fig. 6b shows, an MZI mesh based on the orthogonal matrix transformation was used as a 4 × 4-port universal linear circuit, enabling self-adaptation to implement the desired functions^68^. The same structure shown in Fig. 6c could further automatically undo strong mixing between modes as a mode descrambler^69^. The theoretical analysis for the initialization procedure, training and optical multiple-input multiple-output equalizers was discussed in detail in refs. ^72,101,102^. More generally, the MZI-based orthogonal matrix mesh was theoretically proved to have the ability to analyze and generate multiple modes using self-configuring methods^76^. The concept and architecture are presented in Fig. 6d, where an example of a square grating coupler array is illuminated by the input light. While these self-configuring methods require many built-in optical power monitors, they bring additional loss and rapidly increase the number of monitors with the extension of the network, making both the electronic layout and iterative algorithm quite complex. In 2020, Zhou et al. proposed and experimentally demonstrated a common self-configuring method without any information from the inner structure^103,104^. Figure 6e shows an example of the iteration process, where a switching matrix was self-configured from a random state. The training was finished using the numerical gradient algorithm inspired by deep learning^3^, which is practicable for a general “black box” system. A similar idea was applied for an all-in-one photonic polarization processor chip^105,106^. Other MZI meshes were also reported for multipurpose silicon photonics signal processors, such as a hexagon mesh^107^ and a square mesh^108^.

### WDM-MVMs

**Fig. 7:**
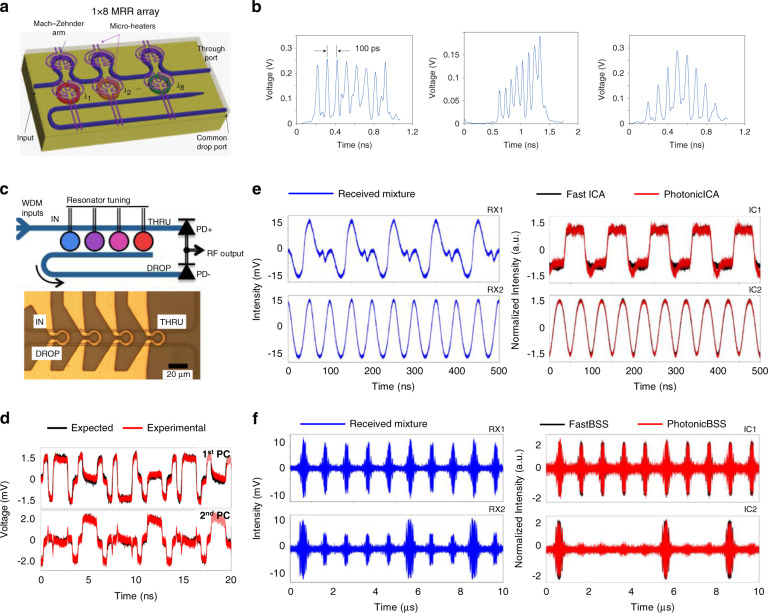
WDM-MVMs for optical signal processing. **a**, **b** On-chip programmable pulse processor employing a cascaded MZI-MRR structure^110^. **c** Microring weight banks^83^. **d** Photonic principal component analysis^87^. **e** Photonic independent component analysis^86^. **f** Radio-frequency blind source separation^88^. **a**, **b** Reproduced from ref. ^110^ with permission of Springer Nature: Nature Photonics. **c** Reprinted with permission from ref. ^83^ © The Optical Society. **d** Reprinted with permission from ref. ^87^ © The Optical Society. **e** Reprinted with permission from ref. ^86^ © The Optical Society. **f** Reprinted with permission from ref. ^88^ © The Optical Society.

The WDM-MVM can be directly executed without any algorithms, benefiting from the one-to-one mapping relation between wavelengths and matrix elements. This correlation makes the WDM methods practicable for wave shaping combined with frequency–time mapping^109,110^. As shown in Fig. 7a, b, a 1 × 8 MRR array was fabricated for on-chip programmable pulse shaping. The spectral shape and width could be tuned by changing the resonant wavelengths of the MRRs. The square-shape transfer function is demonstrated and presented in Fig. 7b. Other shapes, such as an isosceles triangle and a sawtooth triangle, were also verified. Furthermore, the MRR array can be used for MVM, provided that a sum operation on multiple wavelengths is performed, called “microring weight banks”, as shown in Fig. 7c^83^. A balanced photodetector (PD) yielded the sum and difference of weighted signals. The reconfigurability and scalability of the channel count of the MRR weight banks were experimentally demonstrated in ref. ^111^ with a comprehensive theoretical analysis^112^. Different methods of controlling large-scale MRRs for matrix computation were proposed and demonstrated in refs. ^85,113,114^. Afterwards, the microring weight bank was applied for various signal processing methods, such as fiber nonlinearity compensation^115^ and photonic PCA^87^. PCA aims to extract the principal components (PCs) solely based on the statistical information of the weighted addition output. Figure 7d presents an experimental example of the obtained two-channel waveforms of both the 1^st^ and 2^nd^ PCs, evidencing the effectiveness of photonic PCA. The weight bank was further used for photonic ICA to identify the underlying sources that form the basis of the observed data^86^. As shown in Fig. 7e, photonic ICA retrieved the corresponding independent components (ICs) from the received mixture waveforms. By combining the photonic PCA and ICA together, a two-step procedure for a complete photonic BSS pipeline was achieved^88^. The BSS is a powerful technique for achieving signal decomposition with minimal knowledge on either the source characteristics or the mixing process. Figure 7f gives an example of ICs retrieved from mixed radio-frequency waveforms with the BSS technique^88^.

In comparison, coherent MVMs are usually applied in multimode signal processing. The MPLC method can manage massive modes benefiting from the ability of large-scale matrix computation. The main limits are that it is bulky and difficult to refresh with a fast response. The MZI method is easy to integrate, and the functions of the MZI mesh can be autoconfigured since the phase shifters can work faster. However, the scale of matrix computation is limited, and this method can work only for a few modes. Compared with the MZI method, the WDM-MVM method has a more compact footprint, and it is much easier to configure the transmission matrix and apply WDM-MVM for programmable pulse shaping, photonic PCA, ICA and BSS.

## MVMs for optical neural networks

AI technology has been widely used in various electronics industries, such as for deep-learning-based speech recognition and image processing. MVM, as the basic building block of ANNs, occupies most of the computing tasks, such as over 80% for GoogleNet and OverFeat models^116^. Improving the MVM performance is one of the most effective means for ANN acceleration. Compared with electrical computing, optical computing is poor at data storage and flow control, and the low efficiency of optical nonlinearities limits the applications in nonlinear computation^117^, such as activation functions. While it has significant advantages on massively parallel computing through multiplexing strategies of wavelength, mode and polarization^17,90^, extremely high data modulation speeds up to 100 GHz^118,119^. Hence, photonic networks are quite good at MVM. The combination of optical computing and AI is expected to realize intelligent photonic processors and photonic accelerators^120^. In recent years, AI technology has also seen rapid developments in the field of optics.

### MPLC-MVMs

**Fig. 8:**
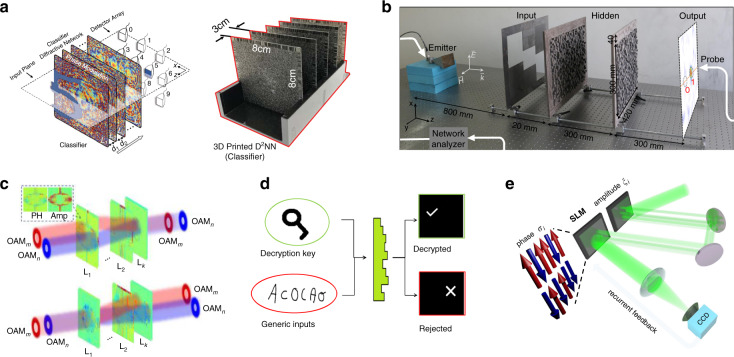
MPLC-MVMs for ONNs. **a** Classification of images of handwritten digits and fashion products^46^. **b** Optical logic operations^54^. **c** OAM multiplexing and demultiplexing^50^. **d** Optical linear perceptrons^56^. **e** Photonic Ising machine^51,123^. **a** Reprinted by permission from AAAS^46^. **b** Reproduced from ref. ^54^ with permission of Springer Nature: Light: Science & Applications. **c** Reprinted from ref. ^50^ with the permission of IEEE Publishing. **d** Reproduced from ref. ^56^ with permission of Springer Nature: Light: Science & Applications. **e** Reproduced from ref. ^123^. with permission of De Gruyter Publishing

**Fig. 9:**
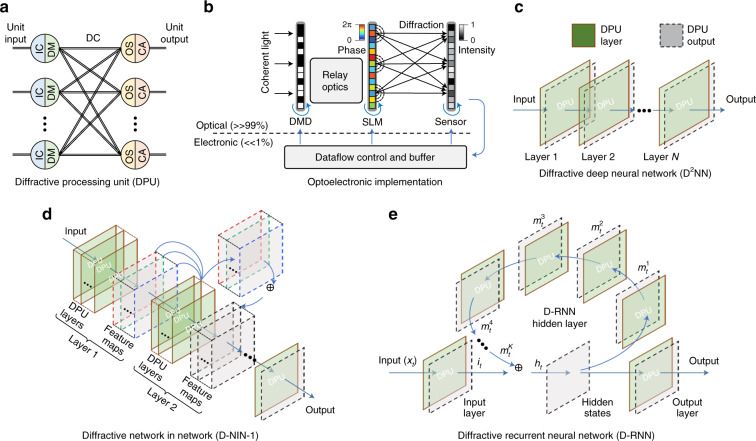
Optoelectronic fused neural computing framework^58^. **a** DPU. **b** Programmable optoelectronic devices to implement the DPU. **c**–**e** Three different types of neural network architectures were constructed, including the D^2^NN, D-NIN-1, and D-RNN. **a**–**e** Reproduced from ref. ^58^ with permission of Springer Nature: Nature Photonics

MPLC, as a supersized MVM method, is an inborn alternative to the ONN. In 2018, Lin et al. presented an all-optical diffractive deep neural network (D^2^NN) architecture to perform machine learning^46^. The schematic diagram is shown in Fig. 8a. Five phase-only transmission masks were used to classify images of handwritten digits and fashion products at the speed of light. Then, a modified D^2^NN based on class-specific differential detection was designed to improve the inference accuracy^47^. The information processing capacity of MPLC was recently discussed in detail by Kulce et al.^121^, proving that the dimensionality of the all-optical solution space is linearly proportional to the number of phase planes. While it may be difficult to train the D^2^NN due to the existence of vanishing gradients, it has been suggested to address this issue by directly connecting the input and output using a learnable light shortcut, which offers a direct path for gradient backpropagation in training^122^. The MPLC-D^2^NN can be applied not only in image identification but also in optical logic operations^54^, OAM multiplexing and demultiplexing^50^, optical linear perceptrons^56^ and Ising machines^52^. As shown in Fig. 8b, the optical logic functions were performed by a two-layer D^2^NN, and different logic operations were output from different ports after the training^54^. The incident wave was physically encoded at the input layer, and then the compound metasurfaces (hidden layer) scattered the encoded light into one of two small designated areas at the output layer, which provided information on the output logic states. On this foundation, multiple logic gates can be further cascaded to enable more complex or customer-defined functionalities. This universal design strategy holds potential in several applications, such as cryptographically secured wireless communication, real-time object recognition in surveillance systems, and intelligent wave shaping inside biological tissues. Figure 8c presents the coupling and separation of OAM modes with the D^2^NN. Here, four plane masks with pixels of 256×256 were designed to couple and separate four OAM modes. The optical machine learning decryptor in Fig. 8d was realized with single-layer holographic perceptrons, which were trained to complete optical inference missions^56^. This decryptor could perform optical inference for single or whole classes of keys through symmetric and asymmetric decryption. The decryptors could be nanoprinted on complementary metal-oxide–semiconductor (CMOS) chips by galvo-dithered two-photon nanolithography (GD-TPN) with axial nanostepping of 10 nm. The high resolution achieved by GD-TPN allowed achieving a small feature size for the holographic perceptrons at near-infrared telecommunication wavelengths and a neuron density of >500 million neurons per square centimeter. MPLC was also applied in a spatial-photonic Ising machine. The principle of a photonic Ising machine with spatial light modulation is depicted in Fig. 8e^51,123^. The spins were encoded into binary optical phases of 0 and π at separated spatial points by an SLM. Intensity modulation was used to set the spin interaction via another SLM. Recurrent feedback from the far-field camera allowed evolution of the phase configuration toward the Ising ground state. It developed a novel hardware with an optics-enabled parallel architecture for large-scale optimizations. A photonic scheme for combinatorial optimization analogous to adiabatic quantum algorithms and classical annealing methods was further studied^52^. More recently, Ruan et al. experimentally evaluated the phase diagram of a high-dimensional spin-glass equilibrium system with 100 fully connected spins under gauge transformation^124^ and synchronously proposed implementing an antiferromagnetic model through optoelectronic correlation computation with 40000 spins for the number-partitioning problem^125^. The nonlinear activation functions for D^2^NN were also proved using laser-cooled atoms with electromagnetically induced transparency^126^. To seek a more general and reconfigurable MPLC-based ONN, an optoelectronic fused computing framework based on optical diffraction was proposed, which supports several kinds of neural networks and maintains a high model complexity with millions of neurons^58^. The principle diagram of the basic diffractive processing unit (DPU) is presented in Fig. 9a, b. A digital micromirror device (DMD) and an SLM were assembled to implement the input nodes, and a CMOS sensor was used to implement the optoelectronic neurons. It consists of large-scale diffractive neurons and weighted optical interconnections, enabling the processing of large-scale visual signals, such as images and videos. Three types of ONNs were configured, including the D^2^NN in Fig. 9c, the diffractive network in network (D-NIN-1) in Fig. 9d, and the diffractive recurrent neural network (D-RNN) in Fig. 9e.

### MZI-MVMs

**Fig. 10:**
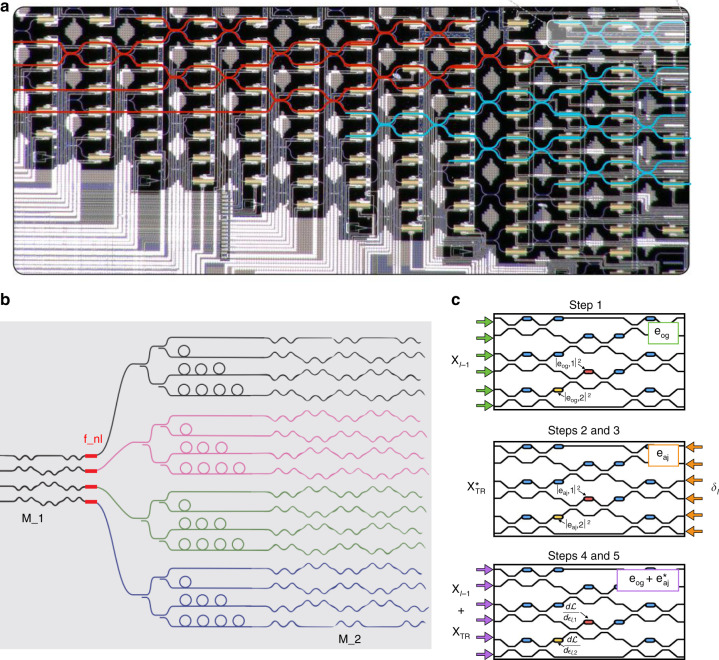
MZI-MVMs for ONNs. **a** Optical feedforward neural network^3^. **b** Optical convolutional neural network^71^. **c** In situ training of an ONN^127^. **a** Reproduced from ref. ^3^ with permission of Springer Nature: Nature Photonics. **b** Reproduced from ref. ^71^ with the permission of the authors. **c** Reprinted with permission from ref. ^127^ © The Optical Society

**Fig. 11:**
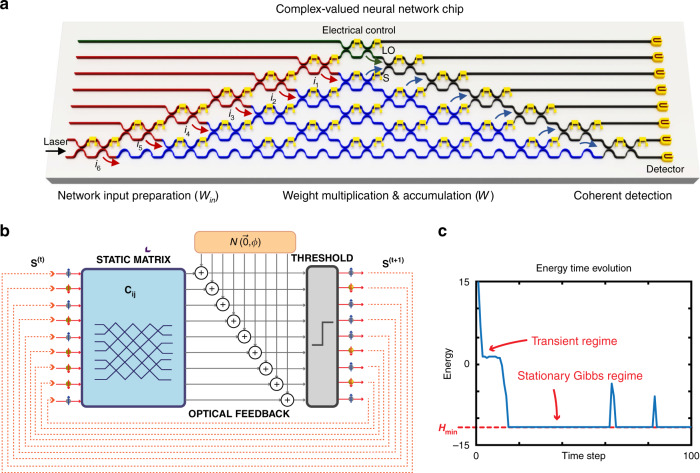
MZI-MVMs for complex-valued ONN and photonic Ising machines. **a** Complex-valued ONN with MZI mesh^77^. **b**, **c** Photonic recurrent Ising machines with MZI mesh^74^. **b** The principle of Ising machines and **c** the energy evolution as a function of time. **a** Reprinted from ref. ^77^ with permission from Springer Nature: Nature Communications. **b**, **c** Reprinted with permission from ref. ^74^ © The Optical Society

Different from MPLC-MVM, the main advantage of MZI-MVM is the potential small size, allowing miniaturized ONN chips. In 2017, Shen et al. proposed a new architecture for a fully optical feedforward neural network, as shown in Fig. 10a^3^. The device, containing 56 programmable MZIs, demonstrated its utility for vowel recognition. It improved the computational speed and power efficiency over advanced electronics for conventional deep learning tasks. Thereafter, an optical convolutional neural network was further proposed. As shown in Fig. 10b, the optical delay lines were implemented with microrings, and the MVM was implemented efficiently in photonic circuits by an MZI mesh^71^. However, the training of these networks was quite difficult and should be followed. Hughes et al. introduced a highly efficient method for in situ training of an ONN. Figure 10c presents a schematic illustration of the proposed method, which uses adjoint variable methods to derive the photonic analog of the backpropagation algorithm^127^. The genetic algorithm was also demonstrated as an efficient method to on-chip train the ONNs^128^. A similar mesh could be expanded to implement a complex-valued neural network^77^. As shown in Fig. 11a, the complex-valued ONN could encode information in both phase and magnitude with MZIs (marked in red). The reference light used for coherent detection was introduced by the MZI in green. The complex-valued weight matrix was implemented with the MZIs in blue. Then, on-chip coherent detection was implemented by the remaining black MZIs. The input preparation, weight multiplication and coherent detection were all integrated onto a single chip, which offered significantly enhanced computational speed and energy efficiency.

In addition to neural networks, efforts have also been made to unleash the potential of these photonic architectures by developing algorithms that optimally exploit photonic fundamental advantages. In 2020, Roques-Carmes and Shen et al proposed the photonic recurrent Ising sampler (PRIS)^75^, a heuristic method tailored for parallel architectures allowing fast and efficient sampling from distributions of arbitrary Ising problems. They later experimentally demonstrated the PRIS by combining electronics and silicon-on-insulator photonics^74^. Figure 11b presents the algorithm iteration of the PRIS. The spin state vector was encoded in the amplitudes of coherent optical signals at the input. The transmission matrix of the MZI mesh was dependent on the problem-specific Ising coupling matrix. The output of the matrix multiplication is noisy with Gaussian perturbation. After several algorithm steps, the energy shown in Fig. 11c could approach the ground state, and then the results of optimization for a specific Ising problem were obtained.

### WDM-MVMs

**Fig. 12:**
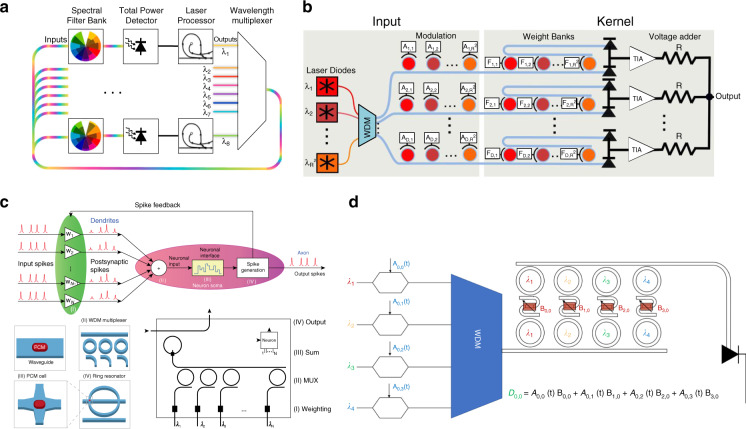
WDM-MVMs for ONNs. **a** Optical broadcast-and-weight network showing parallels with the neural network mode^82,84^. **b** Optical convolutional neural network^92^. **c** All-optical spiking neurosynaptic networks^89^. **d** Photonic dot product engine for machine learning^129^. **a** Reprinted from ref. ^82^ with the permission of IEEE Publishing. **b** Reprinted from ref. ^92^ with the permission of IEEE Publishing. **c** Reprinted from ref. ^89^ with permission from Springer Nature: Nature. **d** Reproduced from ref. ^129^ with the permission of AIP Publishing

In 2014, Tait and his colleagues proposed, for the first time, using MRR arrays as a matrix computation method primitive for photonic neural networks^82^. This work, for the first time, introduced a scalable neural network architecture called “broadcast-and-weight” based on the WDM concept. In this architecture, as shown in Fig. 12a, neural network weights can be continuously tuned to achieve both positive and negative weights analogous to neural weights. In the same work^82^, Tait et al. also first introduced a network design allowing scalable and cascadable ONNs by employing wavelength reuse, followed by an experimental demonstration in 2017^84^, concurrently with other silicon photonic neuromorphic architectures^3^. This network architecture can be applied to construct both feedforward and recurrent neural networks. Microring weighting banks were also employed for optical CNNs^91–93^. In CNNs, as shown in Fig. 12b, the input images are divided into small patches, and these patches are converted into small matrices for MVM operations. In 2019, an all-optical spiking neural network based on phase-change materials (PCMs) was experimentally demonstrated^89^. As shown in Fig. 12c, the input vectors were loaded on beams with different wavelengths and weighted by PCMs. Moreover, the nonlinear activation function was implemented in optics by changing the resonant wavelengths of the microring when the summed power altered the state of PCMs. Figure 12d shows a photonic tensor core for neural networks using PCMs as the reconfiguration elements^129^. The input matrix was modulated by high-speed modulators, and the kernel matrix was loaded using photonic memory based on PCMs. The weighted inputs were then incoherently summed using a photodetector.

**Fig. 13:**
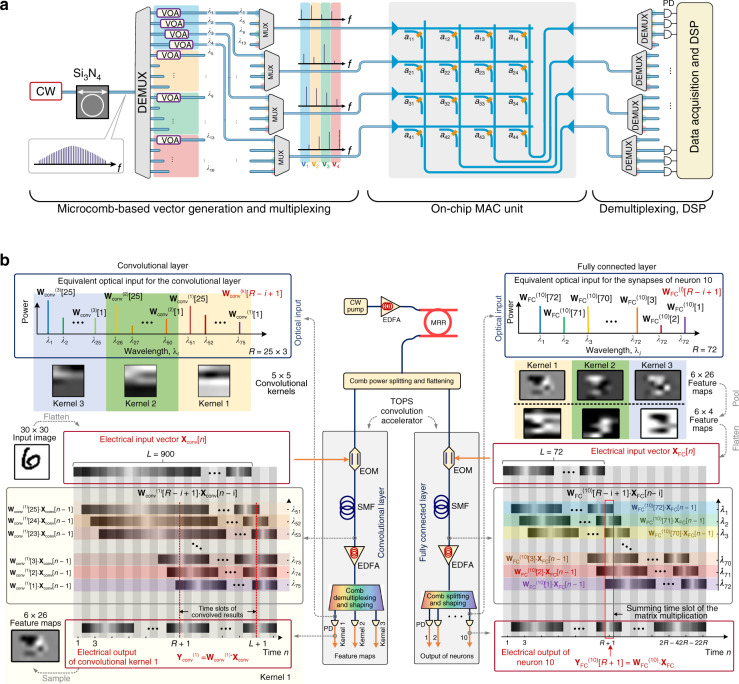
WDM-MVMs for large-scale parallel computing in ONNs. **a** Parallel convolutional processer chip^90^. **b** Photonic convolutional accelerator^16^. **a** Reprinted from ref. ^90^ with permission from Springer Nature: Nature. **b** Reprinted from ref. ^16^ with permission from Springer Nature: Nature

Recently, a convolutional photonic processor with extremely high computing throughputs was demonstrated by exploring different dimensions of light. Feldmann et al. demonstrated a highly parallel convolutional processer using an integrated photonic tensor core, achieving 10^12^ multiply-accumulate operations per second^90^. A conceptual illustration of the photonic architecture is shown in Fig. 13a. Highly parallel MVMs were performed by using multiple groups of wavelengths generated from a soliton-based optical frequency comb. PCMs were applied as nonvolatile actuators, and thus, convolutional processing can be performed with extremely low power. Another photonic convolutional accelerator realized highly parallel computing by utilizing wavelength-and-time interleaving, as shown in Fig. 13b, which achieved up to 10 trillion operations per second^16^. The input data vector was encoded as the intensity of light with an electro-optical Mach–Zehnder modulator (EOM), and then the wavelength-dependent delay achieved by a single-mode fiber (SMF) was used to reshape the signals at different wavelengths. The convolutional operation was performed at the speed of light by summing the powers at presupposed wavelengths after spectral shaping. These works suggest that photonics is coming of age and in some cases can begin to outperform electronic computation.

Regarding neural networks, all three MVM methods can be used in the linear part of neural networks to achieve photonic acceleration. In contrast, MPLC-based ONNs have the most powerful computing ability and can solve classification problems with all-optical methods, but the refresh rate of spatial planes is limited. MZI-based ONNs are reconfigurable for different situations, but their scale is limited, and electronics-aided learning is needed for complex tasks. To date, WDM-based ONNs have a larger scale than MZI-based ONNs, while they are incoherent computations, where differential detection is often carried out in tandem. Table 2 summarizes the performance comparison of state-of-the-art photonic AI accelerators with electronic hardware. In general, photonic computing has obvious advantages in terms of signal rate, latency, power consumption and computing density, and its accuracy is generally lower than that of electrical computing.

**Table 2:** Comparison of different recently demonstrated photonic AI accelerators with electronic hardware

Technology	Signal/frame rate	Computing density (TMACs/s/mm^2^)	Energy/MAC	Latency	Precision (bits)
MPLC with a reconfigurable diffractive processing unit^58^	27,000 FPS^a^	45,000^a^	0.82 fJ MAC^-1^	–	8
Broadcast-and-weight based on WDM^147,170^	~1 GHz	50	2.7 fJ MAC^-1,a^	<100 ps	>5
TeraMAC processor with integrated laser neuron^18^	5 GHz	—	270 fJ MAC^-1,a^	<1 ns	—
Sub-λ Nanophotonics^171^	10 GHz	5000^a^	30.6 aJ MAC^-1,a^	<50 ps	>5
Photonic WDM/PCM in-memory computing^90^	18 GHz	81	17 fJ MAC^-1,a^	250 ps	5
Optical convolutional accelerator based on WDM^16^	63 GHz	—	1.58 pJ MAC^-1^	110 ns (50 ns^a^)	8
Coherent MZI mesh^3^	100 GHz^a^	0.56^a^	30 fJ MAC^-1,a^	<100 ps	8
Google TPU^172^	0.7 GHz	0.58	0.43 pJ MAC^-1^	1.42 ns	8
PUMA^173^	1 GHz	0.29	2.39 pJ MAC^-1^	<10 ns	16
ISAAC^174^	1.2 GHz	0.41	1.9 pJ MAC^-1^	~200 ns	16
Resistor crossbar array (from Mythic)^175^	900 FPS	0.02	0.24 pJ MAC^-1^	<100 μs	8

^a^These specifications can be finished by reequipping the setup with existing technologies.

## Discussion

### Scalability and cascadability of ONNs

There exists a huge gap between the number of weights of ANN in electrical and optical MVMs, for example, the weight parameters of ResNet-50, a popular and widely used deep learning network architecture presented by Microsoft in 2016, have already reached 25 million^130^. To alleviate the issue, one direct and effective solution is to manufacture larger-scale photonic integrated circuit (PIC) chips, and indeed, Lightmatter Inc. has released the world-record 64×64 sized MZI mesh integrated chip ‘Mars’ in 2020, which is capable of performing 4096 MAC operations each time when a new set of input vectors is fed in, and the computing capacity is estimated to be 8 TOPS^95^. Similar to integrated circuits, the PIC chips provide the potential to achieve larger scale and higher integration density as the manufacture technologies improve. Furthermore, optical devices promise massive parallelism by employing WDM and mode division multiplexing (MDM)^17,90^, these parallel operations can be performed in a single physical optical processing core^90^.

The scale-out issue can also be solved by optimizing and improving optical components. For example, the number of neurons can be further expanded utilizing spectrum reuse strategies for the WDM scheme^82^, and the topology structures of neuron cluster, small-world neural network, and interconnected SNN PICs were proposed to build larger-scale on-chip photonic neurons^28^. As the scale of MRR array becomes larger, the controlling technique would be paramount, integrated photoconductive heaters enable control of large-scale silicon photonic MRR array without requiring additional components, complex tuning algorithms, or additional electrical I/Os^131^. The electro-optical modulators using lithium niobate and barium titanate integrated with silicon photonics offer high-speed phase modulation and low operating voltage, making these devices very attractive for PICs designed for photonic computing^132^. The maturity of state-of-the-art silicon nitride platform has enabled low-loss waveguides (<1 dB/m), thus reducing energy consumption and cost compared with current digital electronics, and provided opportunities for the practical application of photonic accelerators to SOI and III–V PICs especially when computation bandwidth and modulation rates continue to increase rapidly^133^. Challenges arise in scaling to larger matrices, since phase shifters in MZI mesh scheme typically consume 10 mW to 20 mW per unit for thermal tuning^134^, and thermal power consumption accumulation for thousands of phase shifter units will deteriorate the competitiveness of the photonic accelerator. Nano-optical-electro mechanical system (NOEMS) technology can be applied to replace traditional thermal phase shifters to reduce the power consuming of maintaining the status of MZIs^135^. Compared to thermal phase shifters, the static power dissipation of NOEMS components is nearly zero because mechanical displacements only require a small amount of energy to move the waveguide back and forth.

To form a scalable neural network, optical neurons should be able to excite with a certain strength to evoke at least an equivalent response in a downstream neuron^82^. To construct a cascadable neuron, the first step is to use an active amplifier, which provides energy gain in the optical or electrical domain^136^. The second step is to improve the efficiency of optoelectronic devices, which can be achieved by enhancing the interaction between the active materials and propagating waveguide mode (i.e., light-matter interaction) with nanoscale devices and novel materials^137–140^. And hybrid integration technology is significant for integrating the low-loss passive silicon or silicon nitride waveguides with the active amplifiers and lasers^141–143^. These promising technologies pave the way for cascadable photonic neurons.

### Activation functions

MVMs and activation functions are two basic elements of perceptrons^94^. Photonic MVMs show significant advantages on signal rate, latency, computing density and power consumption compared to electrical neurons, while photonic activation functions are still not mature. The implementation of photonic neurons relies on the nonlinear response of optical devices. Based on the physical representation of signals inside a neuron, the techniques are divided into two primary categories: optical-electrical-optical (OEO) and all-optical activation functions. OEO neurons convert optical power into an electrical current and then back into the signal pathway. Their nonlinearities manifest themselves in the electrical domain as well as during the EO conversion step, in which lasers^144–146^ or saturation modulators^147,148^ are employed. Using foundry-compatible silicon-on-insulator (SOI) technology, OEO neurons were demonstrated by Tait et al. using a high-speed silicon MRR modulator^147^ and by Williamson et al. with a Mach–Zehnder-type modulator^149^. All-optical neurons depend on semiconductor carriers, reverse saturated absorption, or optical susceptibility, which can be found in a variety of materials^150^. All-optical neuron implementations are thought to be faster than the OEO techniques. All-optical neurons have been proven using optical nonlinearities, such as the carrier effect in MRRs^151–153^ and the alteration of a material state^89,154^. Generally, for different AI applications, activation functions need to be chosen dependent on particular tasks. Due to the weak optical nonlinearity, the resonant devices were used to reduce the threshold and simultaneously enhance the phase sensitivity^89,152^. Huang et al. proposed using multiple coupled cavity devices to optimize different activation functions for different machine-learning tasks^152^, followed by an experimental demonstration^153^. And the microring resonators with PCMs were also demonstrated as effective all-optical activation functions^89^. The active optical devices are also promising candidates for activation functions^144,155–157^. A reconfigurable photonic activation function was also demonstrated using injection-locked Fabry–Perot semiconductor lasers^155^. The neuronlike excitable behavior in a micropillar laser with saturable absorber was experimentally demonstrated by introducing optical perturbations^144^. And the vertical-cavity surface-emitting laser with an embedded saturable absorber was employed as a spiking neuron^156,157^. The semiconductor optical amplifiers were also demonstrated for all-optical activation functions^158–161^.

### Optoelectronic-hybrid AI

The activation function can be realized by using either electronic or photonic methods. The optical activation function is still in the preliminary research stage, and there is no mature scheme since the efficiency of optical nonlinearity is rather low. The realization of an all-optical activation function with a low loss and a high nonlinear effect remains a key issue in the entire optical network. On the other hand, all-optical cascaded ONNs are still difficult to achieve due to the accumulative loss of optical networks. In fact, only ANNs with quite simple structures or without activation functions were all-optical, such as the SNNs with PCMs^89^, reservoir computing using optical amplifiers or passive silicon circuits^31,162,163^, and D^2^NN with passive phase masks^46,47,54^. On the contrary, most previous works of deep ANNs were implemented based on optoelectronic-hybrid hardware^3,16,18,58,90^. Before the all-optical ANNs are mature, especially in optical nonlinear effect and optical cascade, optoelectronic-hybrid AI is a more practical and more competitive candidate for deep ANNs. Therefore, the development of a highly efficient and dedicated optoelectronic-hybrid AI hardware chip system is one of the core research routes of photonic AI.

**Fig. 14:**
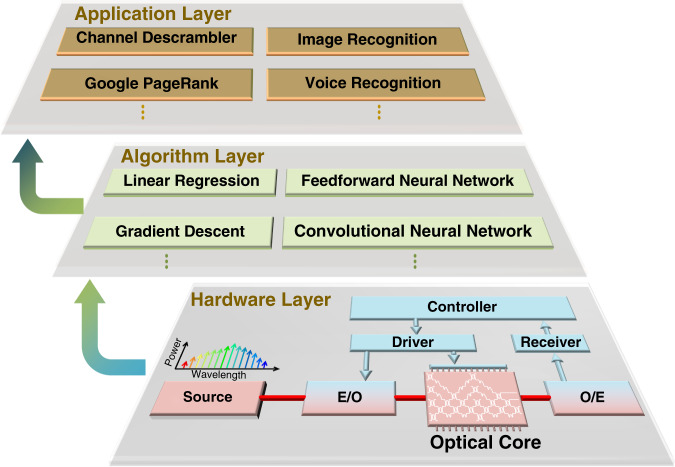
Optoelectronic-hybrid AI. Schematic diagram of the optoelectronic-hybrid AI computing chip framework.

Photonic matrix multiplication has revealed great potential for optical signal processing and AI acceleration. It can greatly reduce the power consumption and signal delay. In the future, the photonic matrix core would be more comprehensive and cover richer functions. Figure 14 shows a possible route for the optoelectronic-hybrid AI computing chip framework. It mainly contains three layers: the bottom hardware layer, the algorithm layer and the top application layer.

Electronic computing has become quite mature, and it has outstanding advantages in terms of data storage and flow control, which are basically unknown for photonic computing. The computing capacity and speed of photons are superior to those of electronic computing, which can be improved by several orders of magnitude^23,164^. By combining the advantages of electronic and photonic systems, the performance in terms of the power consumption, computing capacity, computing speed, etc., can be improved by orders of magnitude compared with that of traditional electronic methods^3,16,58^. The hardware layer is mainly regarded as the photonic AI hardware system built on photoelectric devices. The electronic part of the hardware layer completes the data storage, data write/read, flow control and slight computations. The optical part executes the matrix computation operators, which take up most of the computing tasks^58^.

The algorithm layer is used to develop universal algorithm frameworks for the photonic AI hardware system, such as linear regression and gradient descent^165^, or to develop computing models, such as the feedforward neural network and convolutional neural network^7^. These algorithms can be efficiently executed in the physical layer. Different algorithms can be combined with photoelectric AI hardware depending on the type of problem. For example, the linear regression algorithm is often used in prediction, and logistic regression is often used to solve the problem of binary classification^165^. Neural network algorithms are the most widely used machine-learning methods and can significantly improve deep learning based on text, images, and voice^7^. In addition, based on the activation function, various logical computing functions can be developed as the basic unit of the optoelectronic-hybrid digital computer^166^. The algorithm framework can be learned from the mature AI algorithms of electronic computing, but it should be adjusted appropriately considering the hardware differences.

The application layer is a user-oriented interface based on the entire AI hardware system and algorithm frameworks. Users can develop various applications, such as channel equalization^69,103^, Google PageRank^104^, image recognition^16,90^, and voice recognition^3^. For example, the linear part of the optical computing core can be directly used in image sharpening, smoothing, etc., as well as in all-optical signal processing (such as channel equalization)^167^. Neural network algorithms can be employed for image recognition and voice recognition^3,16,90^. In addition, multiple algorithms can be combined to jointly address optimization and decision issues, such as NP-hard problems and high-speed tracking problems^51,74,168^. An optical computing system based on digital logic can also be built with all-optical or optoelectronic-hybrid logic computing functions^166,169^.

In summary, photonic matrix multiplication has been applied in many areas, such as optical signal processing in optical communications and AI accelerators. Numerous promising applications established based on matrix multiplication computation provide a complementary opportunity to expand the domain of photonic accelerators. We have reviewed the recent progress in photonic matrix multiplication with various methods and applications. A perspective for photonic matrix multiplication was further discussed, which might be extended to an easy-to-operate minicomputer for different photonic accelerator applications.

## Supplementary information

Copyright for Fig.12(a)

Copyright for Fig.12(b)

Copyright for Fig.12(c)

Copyright for Fig.12(d)

Copyright for Fig.13(a)

Copyright for Fig.13(b)

Copyright for Fig.3(a)

Copyright for Fig.3(b)

Copyright for Fig.3(c)

Copyright for Fig.4(b,c)

Copyright for Fig.4(d)

Copyright for Fig.5(a)

Copyright for Fig.5(c)

Copyright for Fig.6(c)

Copyright for Fig.6(e)

Copyright for Fig.7(a,b)

Copyright for Fig.8(a)

Copyright for Fig.8(b)

Copyright for Fig.8(c)

Copyright for Fig.8(d)

Copyright for Fig.8(e)

Copyright for Fig.9
