# Supplementary material for: Photonic matrix multiplication lights up photonic accelerator and beyond
Source: Light Sci Appl. 2022 Feb 3;11:30. doi: 10.1038/s41377-022-00717-8 (PMC8814250; doi:10.1038/s41377-022-00717-8)
Supplement: Supplementary file 2 — Copyright for Fig.12(b) [file 41377_2022_717_MOESM2_ESM.pdf]

IEEE LICENSE
TERMS AND CONDITIONS

Dec 12, 2021

This Agreement between hust -- JIanji Dong ("You") and IEEE ("IEEE") consists of your license details and the terms and conditions provided by IEEE and Copyright Clearance Center.

All payments must be made in full to CCC. For payment instructions, please see information listed at the bottom of this form.

License Number	5206770174563
License date	Dec 12, 2021
Licensed Content Publisher	IEEE
Licensed Content Publication	IEEE Journal of Selected Topics in Quantum Electronics
Licensed Content Title	Digital Electronics and Analog Photonics for Convolutional Neural Networks (DEAP-CNNs)
Licensed Content Author	Viraj Bangari; Bicky A. Marquez; Heidi Miller; Alexander N. Tait; Mitchell A. Nahmias; Thomas Ferreira de Lima; Hsuan-Tung Peng; Paul R. Prucnal; Bhavin J. Shastri
Licensed Content Date	Jan.-Feb. 2020
Licensed Content Volume	26
Licensed Content Issue	1
Volume number	26
Issue number	1
Type of Use	Journal/Magazine
Requestor type	non-commercial/non-profit
I am an IEEE member OR the author of this IEEE content.	member
Format	print and electronic
Portion	figures/tables/graphs
Number of figures/tables/graphs	1
In the following language(s)	Original language
Title of new article	Photonic matrix multiplication lights up photonic accelerator and beyond
Lead author	Jianji Dong
Title of targeted journal	Light: Science & Applications
Publisher	Springer Nature
Expected publication date	Dec 2021
Portions	Fig.5
IEEE membership ID	90314075
Requestor Location	hust<br>wuhan luoyu road<br><br>Wuhan, China 430074<br>China<br>Attn: hust
Billing Type	Credit Card
Credit card info	Visa ending in 7974
Credit card expiration	05/2024
Total	**30.00 USD**

Terms and Conditions

TERMS AND CONDITIONS FOR REUSE OF IEEE MATERIAL SELECTED FOR LICENSING (THE "LICENSED MATERIAL") BASED ON “TYPE OF USE” AND “FORMAT” SELECTED FOR LICENSING BY USER

By clicking "accept" in connection withcompleting this licensing transaction, you, as "User" do agree that the following terms andconditions apply to the use of the material you selected for licensing (the"Licensed Material"), along with the Billing and Payment terms and conditionsestablished by Copyright Clearance Center, Inc. ("CCC"), at the timethat you opened your RightsLink account and that are available at any time at http://myaccount.copyright.com.

Grant of Limited License

IEEE hereby grants to you a non-exclusive, non-transferable worldwide license to use the Licensed Material in the "TYPE OF USE" and "FORMAT" that you outlined in the RightsLink form in connection with this transaction, and as outlined in accordance with the terms and conditions of this Agreement. This license does not include any photography, illustrations or advertisements that may appear in connection with the Licensed Material and does not extend to any revision or subsequent edition in which the Licensed Material may appear.

Types of Use

Complete terms and conditions and "TYPES OF USE" can be found in the License Agreement that will be available to you during the online order process. Certain terms of use in some IEEE licenses may take precedence over and supersede certain rights granted through RightsLink services. IEEE also reserves the right to restrict the types and total number of items that may be reused in any type of publication or medium.For additional information on the types of use available by IEEE and through RightsLink, please refer to http://www.ieee.org/publications_standards/publications/rights/rightslink_usetypes.html .

Authorized Use

The license granted is granted for a one-time use for REPUBLICATION IN THE "TYPE OF USE" and "FORMAT" that you outlined in the RightsLink form in connection with this transaction, to be completed within one (1) year from the date upon which this license is effective, with a maximum distribution equal to the number that you identified in the RightsLink form in connection with this transaction.

Restrictions on Use

All uses not specifically authorized in this license and specified in the options for reusing IEEE Licensed Material available through the RightsLink service are prohibited, including (i) altering or modifying the Licensed Material in any manner, translating the Licensed Material into another language or creating any derivative work based on the Licensed Material; (ii) storing or archiving the Licensed Material in any electronic medium or in any form now invented or devised in the future, except where permission is granted to do so. For additional information on the types of use available by IEEE and through RightsLink, please refer to http://www.ieee.org/publications_standards/publications/rights/rightslink_usetypes.html .

If the Licensed Material is altered or modified in any manner, it must be within the scope of the license granted and it must not alter the meaning of the Licensed Material or in any way reflect negatively on the IEEE or any writer of the Licensed Material. Any use of the Licensed Material in any way that would be considered libelous, defamatory, abusive or obscene, in violation of any applicable law or the proprietary rights of a third party and or used in connection with the advertising or promotion of any product or service is also prohibited.

You agree to use your best efforts to prevent unauthorized use of the Licensed Material.

License Effective Only Upon Payment and Author Approval

The license granted to you is effective only upon (i) receipt of full payment fromyou as provided in CCC's Billing and Payment terms and conditions and (ii) yourhaving obtained the author's approval of your proposed use of the LicensedMaterial as described here.

IEEE Intellectual Property Rights

You agree that IEEE is the owner of all right, title and interest in the Licensed Material and/or has theright to license the Licensed Material, including all copyright rights andother intellectual property rights under United States and international law.

Termination

In the event that you breach any of these terms and conditions or any of CCC's Billing and Payment terms andconditions, the license granted herein shall be terminated immediately. Any useof the Licensed Material after termination, as well as any use of the LicensedMaterial beyond the scope of these terms and conditions, may constitutecopyright infringement and IEEE reserves the right to take any and all actionto protect its rights in the Licensed Material.

Copyright Notice

You must include the following copyright and permissionnotice (WITH DETAILS FILLED IN BY YOU) in connection with the Authorized Use ofthe Licensed Material:

© [Year] IEEE. Reprinted, with permission, from [complete publication information].

Warranty and Indemnity

You warrant that you have all rights necessary to enter into this agreement and hereby indemnify andagree to hold harmless IEEE and CCC, and their respective officers, directors,employees and agents, from and against any and all claims arising out of youruse of the Licensed Material other than as specifically authorized pursuant tothis license.

Limited Warranty and Limitation of Liability

THE RIGHT TO USE THELICENSED MATERIAL IS GRANTED ON AN "AS IS" BASIS AND IEEE MAKES NO WARRANTY, EXPRESS OR IMPLIED WITH RESPECT TOTHE LICENSED MATERIAL, INCLUDING ALL WARRANTIES OF QUALITY, ACCURACY AND/ORFITNESS FOR A PARTICULAR PURPOSE, AND IEEE SHALL NOT BE LIABLE UNDER ANYCIRCUMSTANCES FOR ANY LOSSES RESULTING FROM YOUR RELIANCE ON OR USE OF ANYINFORMATION CONTAINED IN THE LICENSED MATERIAL.

No Transfer or Assignment of License

This license is personal to you and may not be sublicensed, assigned, or transferred by you to any otherperson without IEEE's written permission.

Objection to Contrary Terms

IEEE hereby objects to any terms contained in any purchase order, acknowledgment, check endorsement orother writing prepared by you, which terms are inconsistent with these termsand conditions or CCC's Billing and Payment terms and conditions. These termsand conditions, together with CCC's Billing and Payment terms and conditions(which are incorporated herein), comprise the entire agreement between you andIEEE concerning this licensing transaction. In the event of any conflictbetween your obligations established by these terms and conditions and thoseestablished by CCC's Billing and Payment terms and conditions, these terms andconditions shall control.

Payment Terms

If you would like to pay for this license now, please remit this license along with your payment made payable to "COPYRIGHT CLEARANCE CENTER" otherwise you will be invoiced within 48 hours of the license date. Payment should be in the form of a check or money order referencing your account number and this invoice number. Payments should be sent to the address noted below:

Copyright Clearance Center
29118 Network Place
Chicago, IL 60673-1291

Once you receive your invoice for this order, you may pay by credit card. Additional information is provided to users at the time their credit card order is placed.

For suggestions or comments regarding this order, contact RightsLink Customer Support: customercare@copyright.com or +1-855-239-3415 (toll free in the US) or +1-978-646-2777.

Comments and Questions for IEEE

All comments and/or questions related to RightsLink permission services or comments and questions regarding IEEE licensing policies should be sent to discoverservices@ieee.org. Users may also call Author Support & Content Discovery staff at 732.562.3965.

A copy of both the license and these terms should be retained for your files.

Other Terms and Conditions:

Updated 12/2011 nbd-IEEE

Questions? customercare@copyright.com or +1-855-239-3415 (toll free in the US) or +1-978-646-2777.
