# Supplementary material for: Photonic matrix multiplication lights up photonic accelerator and beyond
Source: Light Sci Appl. 2022 Feb 3;11:30. doi: 10.1038/s41377-022-00717-8 (PMC8814250; doi:10.1038/s41377-022-00717-8)
Supplement: Supplementary file 8 — Copyright for Fig.3(b) [file 41377_2022_717_MOESM8_ESM.pdf]

RightsLink®

Help Email Support

Time reversed optical waves by arbitrary vector spatiotemporal field generation

SPRINGER NATURE

Author: Mickael Mounaix et al

Publication: Nature Communications

Publisher: Springer Nature

Date: Nov 16, 2020

Copyright © 2020, The Author(s)

Creative Commons

This is an open access article distributed under the terms of the Creative Commons CC BY license, which permits unrestricted use, distribution, and reproduction in any medium, provided the original work is properly cited.

You are not required to obtain permission to reuse this article.
To request permission for a type of use not listed, please contact Springer Nature

© 2021 Copyright - All Rights Reserved | Copyright Clearance Center, Inc. | Privacy statement | Terms and Conditions
Comments? We would like to hear from you. E-mail us at customercare@copyright.com
