# Supplementary material for: Photonic matrix multiplication lights up photonic accelerator and beyond
Source: Light Sci Appl. 2022 Feb 3;11:30. doi: 10.1038/s41377-022-00717-8 (PMC8814250; doi:10.1038/s41377-022-00717-8)
Supplement: Supplementary file 10 — Copyright for Fig.4(b,c) [file 41377_2022_717_MOESM10_ESM.pdf]

回复: Copyright permission requests

prjournal@siom.ac.cn
发给 zhouhailongshe@126.com, jjdong, zhangran 2021-12-14 17:06 隐藏信息

发件人: prjournal@siom.ac.cn<prjournal@siom.ac.cn>
收件人: zhouhailongshe@126.com<zhouhailongshe@126.com>
抄送: jjdong<jjdong@mail.hust.edu.cn>, zhangran<zhangran@siom.ac.cn>
时间: 2021年12月14日 (周二) 17:06
大小: 34 KB

Dear Author,

Photonics Research grants you a non-exclusive, non-transferable license to use：

1、Fig. 1 of the following paper:
He Wen, Yuanhang Zhang, Rachel Sampson, Nicolas K. Fontaine, Ning Wang, Shengli Fan, and Guifang Li, "Scalable non-mode selective Hermite–Gaussian mode multiplexer based on multi-plane light conversion," Photon. Res. 9, 88-97 (2021)

2、Fig. 1(d) of the following paper:
He Wen, Huiyuan Liu, Yuanhang Zhang, Peng Zhang, and Guifang Li, "Mode demultiplexing hybrids for mode-division multiplexing coherent receivers," Photon. Res. 7, 917-925 (2019)

These two papers should be properly cited as reference. The figures can only be used in the aforementioned article to be published in Light: Science & Applications. This license is personal to you and may not be sublicensed, assigned, or transferred by you to any other person without *Photonics Research*'s permission.

Thank you!

Best Regards.

Editorial Office, Photonics Research
Department of English Journals, Chinese Laser Press
Shanghai Institute of Optics and Fine Mechanics, Chinese Academy of Sciences
No. 390, Qinghe Road, Jiading, Shanghai 201800, China
CLP Publishing: www.clp.ac.cn
Phone: 86-21-69918198
Email：fangzixuan@siom.ac.cn

发件人： zhouhailongshe@126.com
发送时间： 2021-12-14 14:59
收件人： prjournal
抄送： jjdong
主题： Copyright permission requests

Dear Copyright center of Photon. Res.

We are preparing a review article titled by “Photonic matrix multiplication computing lights up photonic accelerator and beyond” for publication in Light: Science & Applications. And many figures from Photon. Res. journals need to be reprinted by permission of Photon. Res. publishing group. The details of the figures are listed below by Table 1. We state that these figures are only used for this review. We look forward to receiving your permission.

Table 1. The details of the figures.

Figure From papers.	Figure number in literature papers	Journal	Reprinting number in our paper
[1]	Fig. 1.	Photon. Res.	Fig. 4(b)
[2]	Fig. 1(d)	Photon. Res.	Fig. 4(c)

[1] H. Wen et al., "Scalable non-mode selective Hermite–Gaussian mode multiplexer based on multi-plane light conversion," Photon. Res., vol. 9, no. 2, pp. 88-97, 2021/02/01 2021, doi: 10.1364/PRJ.411529.
[2] H. Wen, H. Liu, Y. Zhang, P. Zhang, and G. Li, "Mode demultiplexing hybrids for mode-division multiplexing coherent receivers," Photon. Res., vol. 7, no. 8, pp. 917-925, 2019/08/01 2019, doi: 10.1364/PRJ.7.000917.

Best wishes

Room D503, Wuhan National Laboratory for Optoelectronics (WNLO)
Huazhong University of Science and Technology (HUST)
Luoyu Road 1037, Wuhan 430074, Hubei, P. R. China
Tel:15902760630
Email: hailongzhou@hust.edu.cn； zhouhailongshe@126.com
地址：湖北省武汉市珞瑜路1037号华中科技大学武汉光电国家研究中心
邮箱： hailongzhou@hust.edu.cn； zhouhailongshe@126.com
电话：15902760630
