# Supplementary material for: Photonic matrix multiplication lights up photonic accelerator and beyond
Source: Light Sci Appl. 2022 Feb 3;11:30. doi: 10.1038/s41377-022-00717-8 (PMC8814250; doi:10.1038/s41377-022-00717-8)
Supplement: Supplementary file 13 — Copyright for Fig.5(c) [file 41377_2022_717_MOESM13_ESM.pdf]

CCC RightsLink®

Home | Help | Live Chat | Jlanji Dong

ACS Publications
Most Trusted. Most Cited. Most Read.

Ten-Port Unitary Optical Processor on a Silicon Photonic Chip

Author: Rui Tang, Ryota Tanomura, Takuo Tanemura, et al
Publication: ACS Photonics
Publisher: American Chemical Society
Date: Jun 1, 2021

Copyright © 2021, American Chemical Society

PERMISSION/LICENSE IS GRANTED FOR YOUR ORDER AT NO CHARGE

This type of permission/license, instead of the standard Terms and Conditions, is sent to you because no fee is being charged for your order. Please note the following:

- Permission is granted for your request in both print and electronic formats, and translations.
- If figures and/or tables were requested, they may be adapted or used in part.
- Please print this page for your records and send a copy of it to your publisher/graduate school.
- Appropriate credit for the requested material should be given as follows: "Reprinted (adapted) with permission from {COMPLETE REFERENCE CITATION}. Copyright {YEAR} American Chemical Society." Insert appropriate information in place of the capitalized words.
- One-time permission is granted only for the use specified in your RightsLink request. No additional uses are granted (such as derivative works or other editions). For any uses, please submit a new request.

If credit is given to another source for the material you requested from RightsLink, permission must be obtained from that source.

BACK CLOSE WINDOW

© 2021 Copyright - All Rights Reserved | Copyright Clearance Center, Inc. | Privacy statement | Terms and Conditions
Comments? We would like to hear from you. E-mail us at customercare@copyright.com
