# Supplementary material for: Photonic matrix multiplication lights up photonic accelerator and beyond
Source: Light Sci Appl. 2022 Feb 3;11:30. doi: 10.1038/s41377-022-00717-8 (PMC8814250; doi:10.1038/s41377-022-00717-8)
Supplement: Supplementary file 20 — Copyright for Fig.8(d) [file 41377_2022_717_MOESM20_ESM.pdf]

RightsLink®

? Help | Email Support

Nanoprinted high-neuron-density optical linear perceptrons performing near-infrared inference on a CMOS chip

SPRINGER NATURE

Author: Elena Goi et al

Publication: Light: Science & Applications

Publisher: Springer Nature

Date: Mar 3, 2021

Copyright © 2021, The Author(s)

Creative Commons

This is an open access article distributed under the terms of the Creative Commons CC BY license, which permits unrestricted use, distribution, and reproduction in any medium, provided the original work is properly cited.

You are not required to obtain permission to reuse this article.

To request permission for a type of use not listed, please contact Springer Nature

© 2021 Copyright - All Rights Reserved | Copyright Clearance Center, Inc. | Privacy statement | Terms and Conditions

Comments? We would like to hear from you. E-mail us at customercare@copyright.com
